# The Role of Vimentin Peptide Citrullination in the Structure and Dynamics of HLA-DRB1 Rheumatoid Arthritis Risk-Associated Alleles

**DOI:** 10.3390/ijms26010034

**Published:** 2024-12-24

**Authors:** Cinthia C. Alves, Jaila Lewis, Dinler A. Antunes, Eduardo A. Donadi

**Affiliations:** 1 Department of Medicine, Division of Clinical Immunology, Ribeirão Preto Medical School, University of São Paulo, Ribeirão Preto 14049-900, SP, Brazil; cinthiacalves@alumni.usp.br (C.C.A.);; 2 Department of Biology and Biochemistry, University of Houston, Houston, TX 77204-5001, USA

**Keywords:** molecular dynamic simulation, hydrogen bonds, HLA-DRB1, shared epitope

## Abstract

Citrullination, a post-translational modification (PTM), plays a critical role in rheumatoid arthritis (RA) by triggering immune responses to citrullinated self-antigens. Some HLA-DRB1 genes encode molecules with the shared epitope (QKRAA/QRRAA) sequence in the peptide-binding groove which preferentially presents citrulline-modified peptides, like vimentin, that intensifies the immune response in RA. In this study, we used computational approaches to evaluate intermolecular interactions between vimentin peptide-ligands (with/without PTM) and HLA-DRB1 alleles associated with a significantly increased risk for RA development. Crystal structures for HLA-DRB1*04:01, *04:04, and *04:05 bound to citrullinated peptides (PDB ID: 4MCY, 4MD5, 6BIR) were retrieved from the Protein Data Bank and non-citrullinated 3D structures were generated by mutating citrulline to arginine. The pHLA complexes were submitted to four rounds (50 ns each) of molecular dynamic simulations (MD) with Gromacs v.2022. Our results show that citrulline strengthens the interaction between vimentin and the HLA-DRB1 molecules, therefore impacting both the peptide affinity to the HLAs and pHLA stability; it also induces more intermolecular hydrogen bond formation during MD in the pHLA. Citrulline prevents repulsion between amino acid 71β and the P4-residue of native vimentin. Thus, vimentin citrullination seems to affect pHLA binding and dynamics, which may influence RA-related immune responses.

## 1. Introduction

Rheumatoid arthritis (RA) is an autoimmune disease that targets joint tissues and causes chronic inflammation, hyperplasia of the synovial tissue, and the destruction of cartilage and bone. This leads to swelling, stiffness, and joint pain, as well as joint deformities that can significantly limit individual mobility in daily life [1]. Patients with RA exhibit a range of autoantibodies in their serum and synovial fluid. Notably, antibodies targeting citrullinated proteins or peptides (i.e., anti-citrullinated protein antibodies, ACPA) are highly specific for this condition. ACPAs emerge during disease progression and serve as valuable diagnostic markers, as patients testing positive for ACPA tend to experience a more severe form of the disease [2].

Citrullination is a post-translational modification (PTM) process that converts the amino acid arginine to citrulline in a protein. This process is mediated by a group of enzymes called peptidyl arginine deiminases (PADs) that alter the arginine’s side chain and change the hydrophobicity profile of the protein surface, which can change its structure and function [3]. This modification plays a role in physiological processes such as apoptosis and cellular stress, as well as in pathological processes such as infection. In addition, the RA-autoantigens can also undergo such PTMs during these biological processes. Several proteins in the body can undergo citrullination and are highly expressed in the joints, including vimentin, fibrinogen, alpha-enolase, and type II collagen. Among these proteins, vimentin stands out as a type III intermediate filament protein widely expressed by mesenchymal cells, with critical roles in maintaining cellular integrity, supporting intracellular transport, and acting as a sensor for various stress signals. Its strong presence in inflamed joint tissues makes it a particularly relevant autoantigen in RA, where it becomes a frequent target for autoimmune reactions [4]. These proteins, including vimentin, are targets for ACPAs, triggering an autoimmune response that contributes to the development and progression of the disease [3,5,6].

The human leukocyte antigen (*HLA*) class II loci, particularly the *HLA-DRB1* gene, may be a significant inherited risk factor in ACPA-positive RA [7,8,9]. This gene encodes a class II HLA molecule, composed of two chains of similar size (α and β), and expressed in antigen-presenting cells (APCs). In the context of RA, HLA class II molecules present peptides derived from citrullinated proteins. The recognition of these citrullinated peptide-HLA (pHLA) complexes by CD4 T cells triggers an immune response. Because class II HLA molecules have a wider binding cleft, these molecules can bind peptides of 13 to 18 amino acids in length. Typically, an HLA class II molecule has nine pockets distributed along the length of the cleft. The P1, P4, P6, and P9 pockets are more important than the others, because the side chains that fit into them serve as peptide “anchors” in the binding cleft, and these normally project into the interior of the MHC binding groove [10,11].

A group of alleles at the *HLA-DRB1* locus encode a conserved amino acid sequence (QKRAA, QRRAA, or RRRAA) located within the binding groove, covering positions 70 to 74 of the third hypervariable region on the β1 chain of the HLA-DR molecule [9]. This sequence is referred to as the shared epitope and includes a key residue for pocket P4 (i.e., the residue at position 71β in the binding groove, hereafter referred to as the P4-pocket). The shared epitope sequence is common in high-risk alleles for RA, such as HLA-DB1*01:01, HLA-DRB1*04:01, HLA-DRB1*04:04, HLA-DRB1*04:05, HLA-DRB1*14:02, and HLA-DRB1*10:01 [7,8,9]. These alleles can present both citrulline-modified peptides and unmodified native peptides, accepting either citrulline or arginine as anchor residues for the P4 pocket of the binding groove (hereafter referred to as the P4-anchor). However, only the citrullinated peptides trigger an effective response of antigen-specific CD4+ T cells in RA [12] except for HLA-DRB1*14:02 [13], in which both native and citrullinated peptides showed similar binding affinities, likely due to polymorphisms that alter the P4 pocket. Furthermore, previous studies have shown that polymorphisms near the shared epitope in these alleles are also associated with RA and can influence the binding affinity of citrullinated and native peptides, such as His13βSer in HLA-DRB114:02 and Lys-71β/Arg-71β and Gly-86β/Val-86β in HLA-DRB1*04:01, *04:05, and *04:04 molecules, impacting peptide presentation and T cell responses [13,14]. Interestingly, cigarette smoke exposure has been identified as a significant environmental factor in RA development. Individuals carrying specific *HLA-DRB1* alleles, particularly those with the shared epitope, have shown an increased susceptibility to RA when exposed to cigarette smoke [15]. This gene-environment interaction underscores the complex interplay between genetic predisposition and environmental factors in RA pathogenesis.

Citrullinated vimentin-derived epitopes have been shown to stimulate T cell activation and enhance peptide-HLA (pHLA) binding affinity, especially with RA-predisposing HLA-DRB1 molecules such as HLA-DRB1*04:01, HLA-DRB1*04:04, and HLADRB1*04:05. The availability of experimentally solved high-resolution 3D structures (under 2 Å) of these HLA molecules complexed with vimentin-derived peptides enables accurate modeling of pHLA interactions. Furthermore, experimentally solved three-dimensional structures of HLA-DRB1 molecules complexed with vimentin-derived peptides are already available, thus providing a solid basis for molecular analysis and validation, since these crystals have high-resolution structures (Resolution under 2 Angstroms). This allows the accurate modeling of the pHLA interactions, which helps to obtain a more reliable interpretation of the structural and dynamics changes induced by citrullination and turns vimentin an ideal candidate to study its structural and functional impacts in RA.

In this study, we aimed to evaluate the dynamic and intermolecular interactions involved in the presentation of citrullinated vimentin peptides by HLA-DRB1 molecules, emphasizing alleles associated with RA severity and the role of the P4-pocket in binding citrullinated versus unmodified peptides. Considering that (i) HLA-DRB1 alleles exhibit a shared epitope sequence associated with RA severity, (ii) the P4-pocket residue may interact with the citrullinated P4-anchor in the presented peptides, (iii) vimentin-derived epitopes complexed with RA-predisposing HLA-DRB1 molecules induce T cell activation, and (iv) experimentally solved three-dimensional structures of HLA-DRB1 molecules complexed with vimentin-derived peptides are already available, computational approaches were used to analyze HLA-DRB1*04:01, HLA-DRB1*04:04, and HLA-DRB1*04:05 molecules, bound with either citrullinated or unmodified vimentin peptides. Our work provides insights into the dynamic behavior of citrulline interactions with the P4-pocket and how these amino acid modifications might influence disease outcomes.

## 2. Results

### 2.1. Citrulline Increases the Stability of Vimentin Peptides Displayed by HLA-DRB1

**Figure 1:**
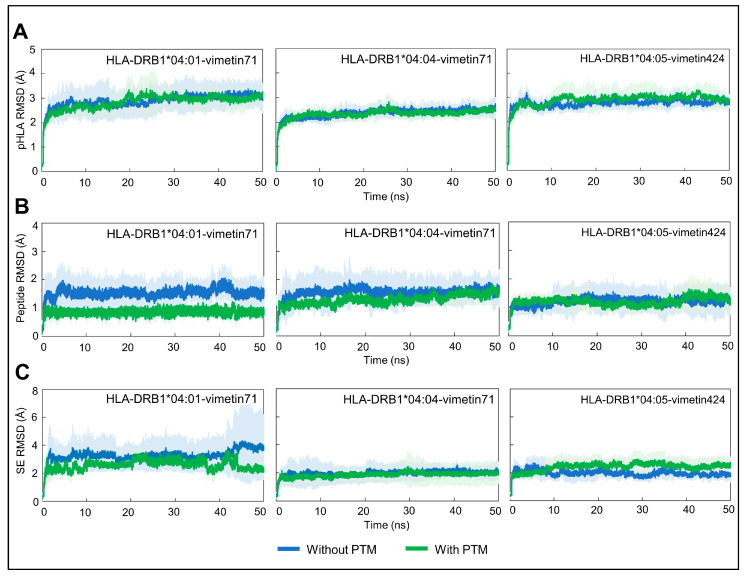
RMSD results of all three pHLA complexes with or without PTMs. RMSD values of pHLA as a whole (**A**), vimentin (peptide) (**B**), and shared epitope (**C**) are plotted. Results are presented as average and standard deviation calculated across the four replicate runs.

Conformational variations were measured by analyzing the RMSD, RMSF, and the alignment between the initial and final structures of the replicated simulations. Figure 1 shows the mean and standard deviation of the RMSD of the protein atoms calculated from the four replicates of each pHLA system (A), for the peptide (B), and for shared epitope (C).

According to the average RMSD results for the pHLAs (Figure 1A), there was not much difference in the range of conformational variability between the complexes regardless of the presence or absence of citrulline, with variation within a range of 2 Å. HLA-DRB1*04:04-vimentin71 displayed the lowest variability, reaching an equilibrium level close to 2 Å in less than 2 ns of production, while the others displayed an average RMSD close to 3 Å across the simulation time.

The average RMSD values calculated for the peptide (Figure 1B) in the pHLA complexes indicate that the presence of citrulline did not significantly alter the conformation of vimentin in the trajectory for most of the analyzed complexes. However, a clear separation was observed for HLA-DRB1*04:01-vimetin71, in which a larger conformational change was observed in the absence of the PTM. This was reflected both in the average RMSD value and the standard deviation across replicates. A lower standard deviation across the replicates was also observed for the citrullinated peptide bound to HLA-DRB1*04:04 and HLA-DRB1*04:05, despite presenting overlapping RMSD averages. Citrulline has a positive impact in the pHLA-DRB1*04:01 stability and dynamics, which was not observed for the other molecules, with and without PTMs.

In agreement with these results, a greater conformational change over time was also observed for the region of the shared epitope in the HLA-DRB1*04:01-vimentin71 complex (Figure 1C), in which the pHLA with the PTM showed lower RMSD average and lower standard deviation as compared to the pHLA with the native peptide. Although the difference in RMSD average is small (e.g., approximately 1 Å), the citrullinated pHLA showed a much higher standard deviation compared to native pHLA, reflecting more conformational differences occurring amongst replicates. It is important to note that the same impact was not observed for the other HLA molecules.

**Figure 2:**
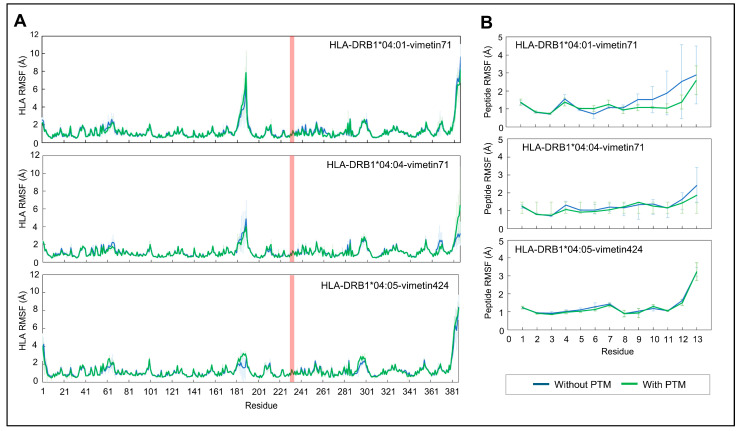
RMSF results (protein group) of pHLA complexes with or without PTMs. RMSF values of HLA (**A**) and vimentin (peptide) (**B**) were plotted. Residues numbered from 1 to 89 belong to HLA-DRA1, 190 to 387 belong to HLA-DRB1, and 230–235 belong to shared epitope in the HLA (**A**) plots. The red box indicates the shared epitope positions for each pHLA system. The results consist of average and standard deviation calculations from the four replicate runs.

The RMSF is used in molecular dynamic simulations (MD) to quantify the local fluctuations and variations in residue positions over time, and was calculated for either the HLA (Figure 2A) and the peptide (Figure 2B), for all the studied pHLAs. As observed in Figure 2A, no significant difference was found between HLA molecules presenting either the native or the citrullinated vimentin peptides, indicating there are similar fluctuation patterns. In general, the RMSF values above 3 Å are predominantly located in terminal regions and loops of the molecules, which are expected to display greater flexibility and dynamics. The HLA-DRB1*04:01-vimetin71 complex showed higher fluctuation in the C-terminus region of the peptide, in comparison to citrullinated counterpart (Figure 2B).

**Figure 3:**
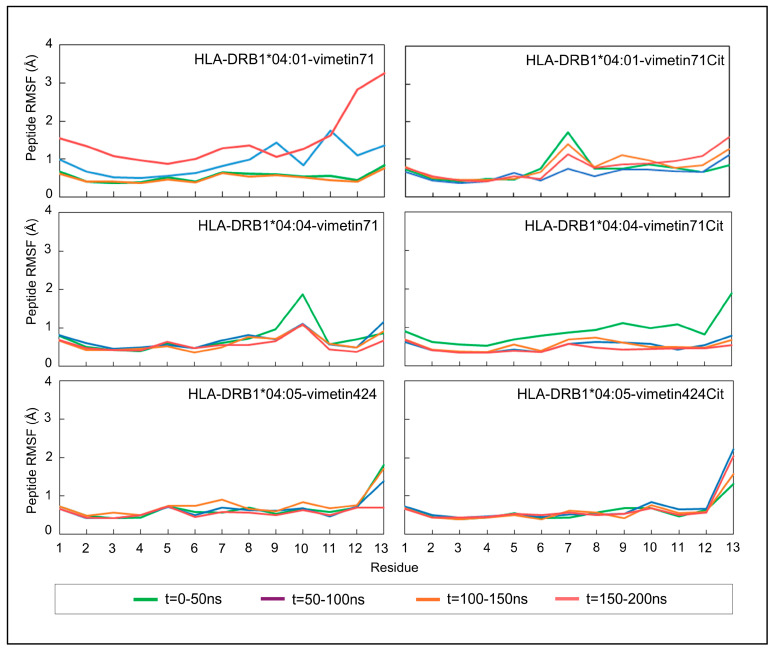
RMSF (protein group) results per replicate for the pHLA complexes, with or without PTMs.

To better explore the impact of the citrulline on the peptides’ dynamics and stability, the RMSF was also calculated per replicate in the studied systems. This analysis allowed for the comparison of the fluctuations and flexibility of each molecule, therefore enhancing interpretability and providing deeper insights into the dynamic behavior of these complex systems amongst replicates. The RMSF results for the replicates, calculated for the alpha carbon (CA) atoms of the peptide, are shown in Figure 3.

The highest conformational variations between the native and citrullinated peptides were observed in the pHLA-DRB1*04:01 complex, as shown in Figure 3. This difference is more pronounced in the C-terminus region of the peptide, as discussed previously. In addition, the citrulline appears to change the backbone structure of a neighboring residue, located at position 7 of the peptide. The other two pHLAs presented conformational differences of less than 1 Å for the RMSF results, with or without PTM, suggesting that the peptide maintained a stable conformation with far less conformational variation during simulated periods. However, the citrulline appears to stabilize the backbone structure of the amino acid at position 10 of the peptide, in contrast to the native pHLA bound to the HLA*DRB1*04:04 allele.

Visual inspection of the pHLA complexes was performed to evaluate the conformational changes that occurred during the production stage of the MD simulation. Replicated runs were concatenated into a single, long trajectory, and the atomic coordinates of the molecular complexes were extracted at specific times from the trajectory as well. These structures were superimposed onto the initial structure (seen in the first frame) using the PyMol v.2.4 software.

**Figure 4:**
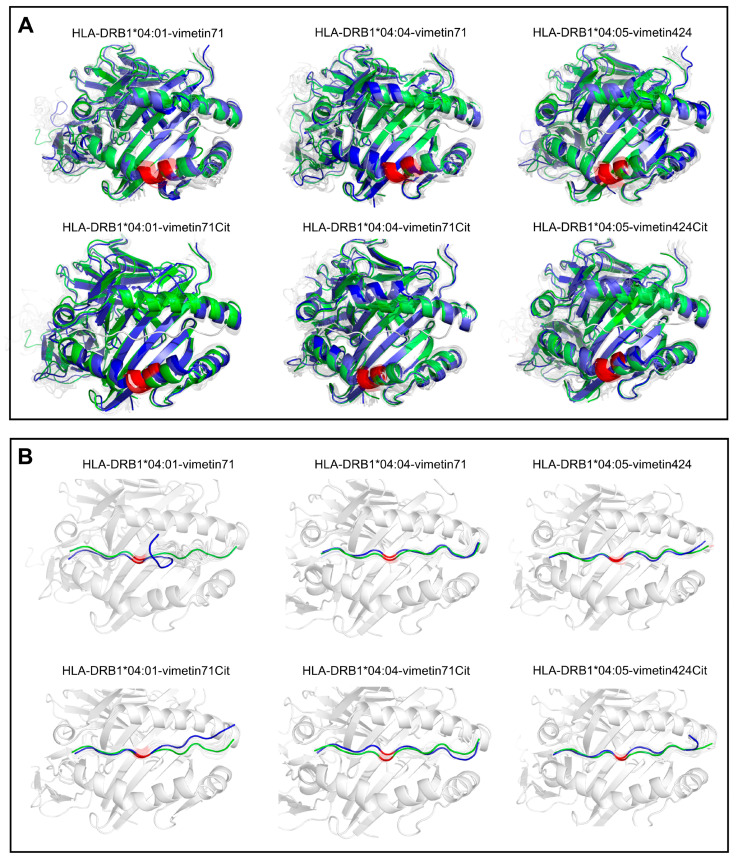
MD trajectory visualization for the structural superposition representation for (**A**) HLA binding cleft and (**B**) vimentin peptide. Representative conformations of the concatenated trajectory are highlighted in green (first frame) and blue (last frame); conformations were extracted in 10 ns intervals across the concatenated trajectory. The red highlighted portions represent either the shared epitope region in the HLA molecule or the peptide ARG/CIT residues in position 6.

Figure 4 shows the structures captured at the determined times for each trajectory. The analysis of pHLA complexes revealed two key findings: (i) the secondary structures of HLAs remained stable throughout the simulation, exhibiting transient oscillations in certain regions without unfolding of the binding cleft (Figure 4A), and (ii) the peptide demonstrated reduced mobility within its C-terminus for the HLA-DRB1*04:01-vimetin71 complex in comparison to the native pHLA (Figure 4B). This observation aligns with the earlier findings on RMSD analysis, highlighting the positive impact of citrullination in the peptide C-terminus stability, especially to the HLA-DRB1*04:01 allele.

### 2.2. Citrullination Enhances the Binding Affinity Between Vimentin Peptides and HLA-DRB1 Molecules

**Figure 5:**
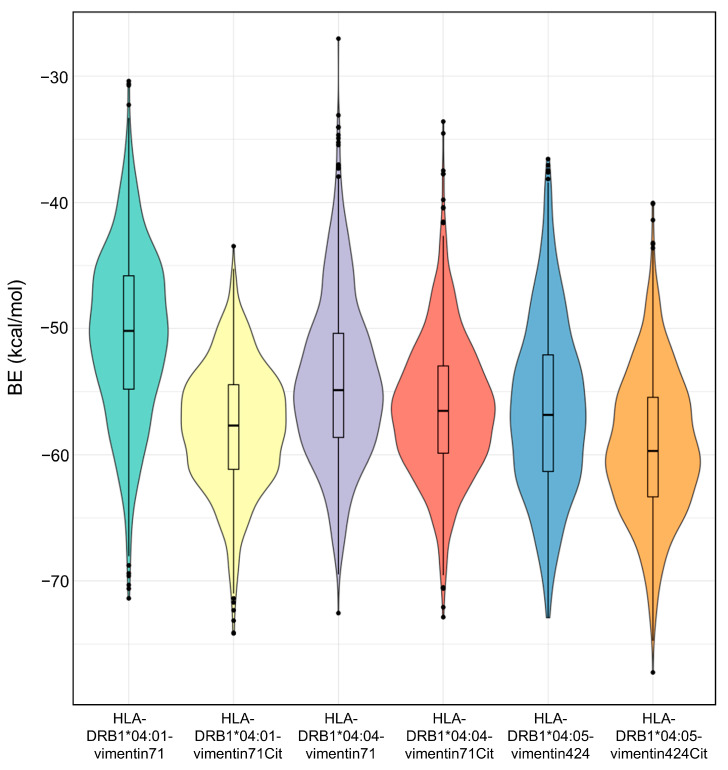
pHLA BE calculations for each pHLA system. Binding energies were calculated for 251 pHLA conformations extracted from each trajectory replicate.

The binding energies (BE) for the pHLAs were calculated with AutoDock4 [16,17] to estimate the impact of citrullination on the peptides’ binding to the HLA molecules. The BE results are shown in Figure 5 and were calculated for an ensemble of conformations extracted from the MD trajectories. The BE scores of pHLA complexes bound to citrullinated peptides appear to be better than their non-citrullinated counterparts in all pHLAs (i.e., more negative values indicate a stronger binding). When comparing the different HLA-DRB1 molecules, HLA-DRB1*04:01 exhibits the biggest BE difference between citrullinated and non-citrullinated vimentin ligands, while HLA-DRB1*04:05 shows the best overall BE score.

### 2.3. Citrullinated pHLA Complexes Present a Higher Prevalence of Hydrogen Bonds over Time

**Figure 6:**
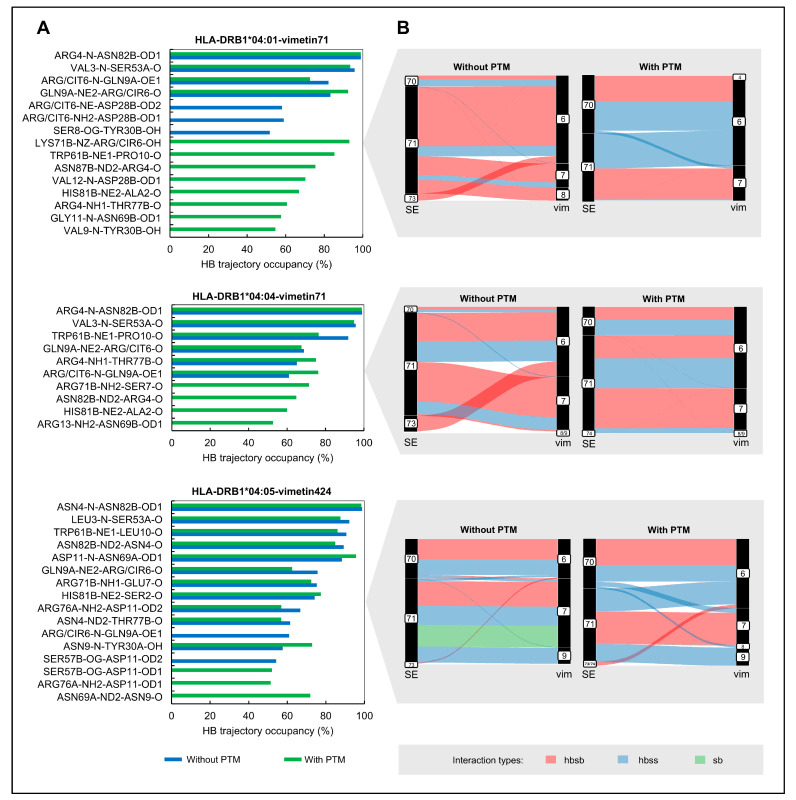
Intermolecular contacts for each pHLA system. (**A**) The first plot on the left side shows intermolecular hydrogen bonds with more than 50% prevalence observed during MD simulations for the HLA-DRB1*04:01, HLA-DRB1*04:04, and HLA-DRB1*04:05 molecules (the residues paired with the amino acid number, chain, and atom shown on the x–axis refer to the amino acids and atoms donor–amino acid and atom acceptor). (**B**) The second plot on the right side (grey boxes) presents intermolecular contacts network between the shared epitope (SE) amino acids of the HLA-DRB1 and vimentin (vim) for each pHLA, with and without PTM (interaction types detected: hbsb: hydrogen bonds side chain to backbone; hbss: hydrogen bonds side chain to side chain; sb: salt-bridge).

Hydrogen bonds (H-bonds) are crucial for maintaining stability and specificity in molecular interactions. Our next step was to evaluate the existence and prevalence of H-bonds over time. Figure 6A shows the H-bond interactions between peptide-ligand and the HLA-receptor that were observed in over 50% of the MD simulations. In addition, to better understand intermolecular contacts distribution in the pHLA complexes regarding the presence of the PTMs, we also calculated other non-covalent contacts between HLA-DRB1 molecules and the presented peptides. Figure 6B shows the calculated intermolecular contacts between the shared epitope amino acids and peptides of the pHLAs using the GetContacts library.

In general, the pHLA complexes with PTMs have more residues with H-bond interactions above the 50% prevalence cutoff (Figure 6A). For the HLA-DRB1*04:01 molecule, we observed a total of 15 H-bonds, with a remarkable distribution, where 8 are exclusive to citrullinated complexes, three are exclusive to non-citrullinated complexes, and 4 are shared between both. For the HLA-DRB1*04:04 molecule, of the 10 hydrogen bonds identified, more than half are shared between the two types of complexes, with and without PTMs. The HLA-DRB1*04:05 molecule had the highest number of shared H-bonds (11 out of 16). The amount of shared H-bond interactions between citrullinated and native pHLAs suggests that citrullination alters the pattern of H-bonds formation between molecules over time, with a more pronounced effect to the HLA-DRB1*04:01 molecule.

In addition, we observed a high prevalence of H-bonds between the amino acids of the shared epitope and the peptide for most molecules (Figure 6B). However, the distribution of these interactions varied significantly between citrullinated and native pHLAs. In addition to the observed non-covalent bonds variation between the amino acids of the shared epitope with the peptide, it is important to note that the influence of citrulline extends to other regions and residues along the HLA molecule (see supplementary Figures S1–S6).

### 2.4. Native pHLAs Display Repulsive Interactions Between Amino Acid 71β Within the Shared Epitope of HLA-DRB1 and the P4-Anchor of Vimentin

**Figure 7:**
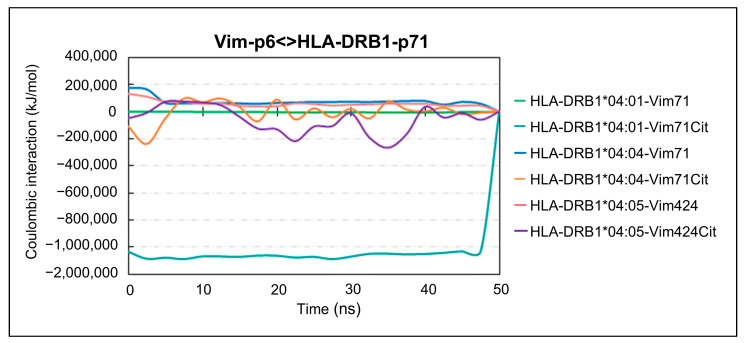
Coulombic energy interaction. Coulombic energy interaction was calculated between the amino acid at position 6 (Vim-p6) of the vimentin and the amino acid at position 71 of the shared epitope in the HLA-DRB1 molecule (HLA-DRB1-p71).

Given that amino acid 71β of the shared epitope contributes to the formation of the P4 pocket of the HLA-DRB1 binding cleft and interacts with the residue of the peptide that is subject to citrullination (i.e., P4-anchor), we computed Coulombic interactions to further investigate the intermolecular forces between these amino acids. This analysis can indicate if the interactions between these residues are mainly attractive or repulsive. Figure 7 shows the Coulombic energy interaction calculated between the P4-anchor of the vimentin (Vim-p6) and the P4-pocket residue (HLA-DRB1-p71), for citrullinated and native pHLAs.

As illustrated in Figure 7, the pHLA complexes involving native peptides exhibited energy values that imply a propensity for repulsion between the P4-pocket residue and the P4-anchor of vimentin. This tendency was particularly pronounced in the HLA-DRB1*04:04-vimentin71 and HLA-DRB1*04:05-vimentin424 complexes, which displayed energy values ranging from 100,000 to 200,000 kJ/mol throughout the trajectory. In contrast, when considering citrullinated peptides, the HLA-DRB1*04:01-vimentin71Cit complex demonstrated energy levels below −100,000 kJ/mol, indicating a robust attractive Coulombic interaction is occurring.

These findings are further supported by the visual analysis of the trajectory conducted with VMD (See Videos S1–S3 in Supplementary Material). It can be observed that complexes containing citrullinated peptides demonstrate a reduction in flexibility for both the P4-anchor of vimentin and the P4-pocket residue of the HLA molecule. The presence of P4-arginine in the peptide and an arginine in position 71 at the shared epitope of HLA-DRB1*04:04 and HLA-DRB1*04:05, results in a repulsion interaction where its side chain is oriented in an opposite position to the other. This effect is mitigated by the substitution with P4-citrulline, which can significantly influence the accommodation of the peptide in the HLA binding groove. Furthermore, there is a tendency for the citrulline’s side chain in the P4-residue of the peptide to project out of the HLA binding groove. This orientation contrasts with the arrangement observed in native peptides and may have implications in antigen presentation and subsequent CD4+ T cell immune response.

## 3. Discussion

Citrullination is a PTM that exacerbates RA severity by altering joint proteins. This is more pronounced in RA patients exhibiting shared epitope-positive-*HLA-DRB1* molecules which results in an intensive immune response to citrullinated peptides. Using MD simulations, we demonstrated how citrulline affects the dynamics and stability of different pHLA complexes. We focused on the HLA-DRB1*04:01, HLA-DRB1*04:04, and HLA-DRB1*04:05 molecules, bound to the vimentin66-78 or vimentin419-431 peptide-ligands, with or without citrullination of the P4-anchor. Our findings reveal that citrulline establishes a distinct network of non-covalent bonds with the receptor, leading to enhanced peptide binding affinity and pHLA stability. These insights contribute to a deeper understanding of the molecular basis of RA, potentially paving the way for more targeted therapeutic approaches in the future.

The protocol adopted in this study consisted of carrying out multiple replicates of “short” molecular dynamics simulations (e.g., four trajectories of 50 ns each), to allow the generation of statistically robust results and explore the differences in intermolecular interactions and stability for pHLA complexes in their native and citrullinated states. It has been well documented in previous work that the analysis of multiple shorter simulations of protein-ligand complexes provides more reliable results than single long trajectories [18,19]. Since we were focused on the analysis of intermolecular interactions (e.g., non-covalent bonds) and large conformational changes are not expected for pHLA complexes in solution, we considered that the simulation time of 50 ns was more than sufficient to explore the dynamics of the interactions between the HLA shared epitope and the peptide-ligands, with or without PTMs. In addition, observing that the secondary structures of the HLAs remained stable throughout the simulation further corroborates that the overall structure of the HLA binding clefts did not undergo significant unfolding or structural changes, which could indicate simulation artifacts or impairment of antigen-presenting function in these systems. Citrullination seems to further stabilize the global structure of peptides displayed by the HLA-DRB1 molecule. This is particularly evident in the C-terminus of the peptide in the HLA-DRB1*04:01 molecule, which is consistent with a positive impact of the PTM on the stability and dynamics of the pHLA during the simulation period.

Citrullination has also been observed to enhance the binding affinity between peptides and HLA-DRB1 molecules, as compared to their native counterparts [20]. The HLA-DRB1*04:05 molecules displayed notably stronger and more stable binding with citrullinated pHLAs. Nevertheless, the HLA-DRB1*04:01 molecule demonstrated the most substantial difference in BE calculations against its non-citrullinated counterpart compared to other molecules. Our findings align with experimental studies employing inhibitory binding assays, in which vimentin self-antigen peptides containing the citrullinated P4-anchor residue were evaluated. This is the case of the peptides vimentin66-78 and vimentin419-431 which display an increased binding affinity when bound to the HLA-DRB1*04:01 molecule [14,21,22]. HLA-DRB1*04:05 also showed higher binding affinity to the citrullinated P4-anchor residue in vimentin419-431 compared to other HLA molecules. However, it’s worth noting that the native HLA-DRB1*04:05-restricted complex also displays a higher binding affinity. This phenomenon may be attributed to the D57S polymorphism, in the P9 pocket of HLA-DRB1, interacting with a negatively charged amino acid in the peptide. The HLA-DRB1*04:05 has a serine in this mutation site and its peptide has an aspartic acid that interacts with the serine 57β, differing from the other two molecules [14]. This observation shows that the pockets beyond the P4 position may exert an influence on peptide binding, which is impacted by the polymorphism within the HLA binding groove. According to Sidney et al. (2017) [23], the impact of citrullination depends on the specific peptide and the HLA allele. Citrullination contributes significantly to the binding of specific peptides, such as vimentin, in the context of HLA-DRB1 shared epitope RA-predisposing alleles, but this is not a consistent effect across all peptides. The effect of citrullination is peptide-specific, indicating that while it enhances binding in some cases, it does not do so universally across all HLA-DRB1 molecules [23]. Furthermore, citrullination influences antigen processing and presentation by HLA class II molecules, which generates a unique citrullination-dependent peptide repertoire. This repertoire is composed of both native and citrullinated sequences and exhibits different binding affinities to HLA-DRB1 alleles [24].

A quantitative analysis of the H-bond prevalence over the simulated time indicated a higher number of bonds occurring exclusively in citrullinated pHLAs, especially in the HLA-DRB1*04:01 molecule. These results may indicate greater specificity (32) as suggested by the consistent formation of specific H-bonds over time, which could stabilize peptide-MHC interactions and potentially enhance antigen presentation, thereby influencing immune recognition and response. Previous studies have demonstrated that citrullination in self-antigens leads to an enhanced effector response in CD4+ T cells in patients with RA compared to healthy individuals. This response is specifically restricted to the *HLA-DRB1* alleles [22,25,26]. In addition, a qualitative analysis of the total number of non-covalent bonds in the trajectory reveals that citrulline may have a distinct impact on non-covalent contacts when compared to the native peptides, suggesting that citrullination alters non-covalent binding distribution in pHLA complexes. This difference in the contacts between pHLAs may have implications for self-antigen recognition by CD4+ T cells in the immune response.

The analysis of Coulombic interaction energies between HLA-DRB1 and vimentin peptides provides crucial insights into the influence of citrullination on pHLA binding dynamics. Our investigation revealed that native pHLA complexes tend to display repulsive interactions between amino acid 71β within the shared epitope of HLA-DRB1 and the P4-residue of vimentin over time. In stark contrast, a significant reduction in repulsive forces was observed for the citrullinated peptides, particularly for the HLA-DRB1*04:01 molecule. In fact, an attractive interaction was observed for these peptides, indicative of enhanced binding affinity, as previously observed. Indeed, the shared epitope located within HLA-DRB1 creates an electropositive P4 pocket with a positively charged residue at position 71β. This favorable electrostatic environment facilitates the binding of autoantigen-derived peptides with polar or acidic residues, while discouraging the binding of peptides with a P4-arginine due to electrostatic repulsion [13,14,22]. Notably, the HLA-DRB1 molecules investigated in this study exhibit polymorphism at the 71β residue within the shared epitope. More specifically, HLA-DRB1*04:01 features a lysine substitution, whereas HLA-DRB1*04:04 and HLA-DRB1*04:05 contain arginine at this position. Both lysine and arginine carry a positive charge that effectively accommodates citrulline in the P4-anchor of the peptide [14]. However, arginine possesses a longer and more flexible side chain in comparison to lysine, which may impair an effective orientation for interaction with citrulline. This structural difference may contribute to the observed fluctuations in energy values over time for the HLA-DRB1*04:04 and HLA-DRB1*04:05 molecules. In contrast, the HLA-DRB1*04:01 molecule displayed consistently strong attractive interaction between Lys71β and citrullinated P4-anchor. Despite the fluctuations observed in the HLA-DRB1*04:04, studies have demonstrated that citrullinated peptides presented by this molecule in RA patients provoke strong responses by CD4+ T cells, whose antigen-specific frequencies strongly correlate with disease activity [27]. This suggests that though the binding dynamics of HLA-DRB1*04:04 may differ from other alleles, they are sufficient to drive significant expansion of antigen-specific CD4+ T cells and thus are critical to the development of autoreactive immune responses.

In conclusion, the citrullinated P4-anchor residue shows a stable conformation over time for the HLA-DRB1*04:01, HLA-DRB1*04:04, and HLA-DRB1*04:05 shared epitope molecules, and the citrulline residue in the peptide accommodates and favorably interacts with the 71β amino acid of the shared epitope throughout the trajectory. Despite this, citrullination seems to alter the nature of the intermolecular forces at the pHLA interface, which potentially affects the stability and binding of the HLA-DRB1 RA-predisposing molecules bound to a vimentin peptide. Of all the studied molecules, the impact is most pronounced for the HLA-DRB1*04:01 molecule. This distinct behavior of the HLA-DRB1*04:01 molecule may be attributed to the Lys71Arg polymorphism within the shared epitope, which can alter the dynamics and the specificity of the interactions between the vimentin peptide and the HLA molecules, as previously discussed. Notably, Lys71Arg in the shared epitope can induce conformational changes in citrullinated peptides, directly affecting their structure and leading to the loss of critical interactions with the TCR [28]. This conformational shift, as reported by Lim et al., (2021), can decrease the response of T cells to pHLA complexes. Specifically, the presence of Arg71β disrupts the contact with Asp109β in the CDR3β loop of the TCR, which modifies the conformation of the P7-anchor in the peptide and thereby diminishes the immune response of autoreactive T cells in RA. Note that our study offers an atomic analysis of pHLA interactions that are hard to capture experimentally, but they rely on computational methods and scoring functions which have limitations. Therefore, these in silico insights should be used to complement the interpretation of existing experimental data and to drive further experimental validation. Taken together, these findings highlighted the intricate interactions between citrulline and the P4 pocket on the shared epitope HLA-DRB1 molecules and their impact in the entire pHLA structure, illustrating how these interactions can influence RA disease outcomes.

## 4. Materials and Methods

### 4.1. pHLA 3D Structures

The co-crystal structures of the RA-risk-associated HLA-DRB1*04:01, HLA-DRB1*04:04, and HLA-DRB1*04:05 molecules bound with a 13-mer citrullinated vimentin peptide-ligand were retrieved from Protein Data Bank (PDB) [29], with PDB codes 4MCY (2.30 angstroms, Å, of resolution) [22], 4MD5 (1.65 Å of resolution) [22], and 6BIR (2.30 Å of resolution) [14], respectively. Missing residues in pHLA complexes were added with the PDB reader tool on CHARMM-GUI server [30]. These reference crystals were also used to create the pHLAs with the respective native peptides, by replacing the citrulline residue in the peptide with an arginine, using the Mutagenesis tool on PyMol v.2.4 software [31]. These pHLA complexes were named according to the presence of the arginine or citrulline as the P4-anchor in the vimentin residue which interacts with the P4 pocket in the shared epitope of the HLA molecules, as highlighted in Table 1.

### 4.2. Molecular Dynamic Simulations (MD)

The MD procedure of pHLA complexes were performed using the GROMACS v.2022 package. The simulations for the native pHLAs were performed using the all-atom CHARMM-27 force field [32]. For citrullinated pHLAs, the same force field was used with the parameters and topology referring to the citrulline [33,34] added to the force field. A cubic box was established, encompassing the structure with a 1.5 nanometer (nm) liquid layer, employing periodic boundary conditions. Sodium (Na^+^) and chloride (Cl^−^) counter-ions were introduced to neutralize each system, reaching a final concentration of 0.15 mol/L. Temperature and pressure coupling were regulated using the v-rescale algorithm (with a time constant of τ-t = 0.1 ps) and the Parrinello–Rahman algorithm (with a time constant of τ-p = 2 picoseconds, ps), respectively. The van der Waals and Coulomb interactions were computed with a cutoff value of 1.2 nm, utilizing fast particle-mesh Ewald electrostatics.

The MD simulations adhered to a previously outlined protocol [35,36]. In summary, the simulation’s production phase followed three energy minimization (EM) steps and eight equilibration (EQ) steps. The initial EM step utilized the steepest-descent algorithm with position restraints on the heavy atoms of the substrate (5000 kJ^− 1^ mol^− 1^ nm^− 1^), permitting solvent relaxation only. The second step employed the same algorithm without restraint, while the third utilized the conjugate-gradient algorithm without restraint. The EQ phase commenced at 310 K for 300 ps, with position restraints on the substrate’s heavy atoms (5000 kJ^− 1^ mol^− 1^ nm^− 1^), facilitating solvation layer formation. Subsequently, the temperature was lowered to 280 K, and position restraints were gradually eased. The temperature was then incrementally raised back to 300 K. These EQ steps constituted the initial 500 ps of each MD simulation. Moreover, each system underwent four replicates of MD simulation with the same protocol, each running for 50 ns at a constant temperature of 300 K without restraint.

### 4.3. Trajectory Analysis

At the end of the simulations, a comprehensive analysis of the trajectories was carried out using GROMACS analysis tools. This analysis included an evaluation of structural variations within the pHLAs throughout the MD trajectories. To quantify these structural changes, Root Mean Square Deviation (RMSD) and Root Mean Square Fluctuations (RMSF) were employed as key metrics. The results were presented as mean values and their corresponding standard deviations derived from the four replicates of each pHLA system. The Coulombic potentials (Coul-SR, Short-range Coulombic Interactions) were also calculated to study the interaction energy between P4-pocket and post-translational modified/unmodified P4-anchor in the peptide during trajectories of the first replicate of each system.

The replica trajectories were concatenated in a unique trajectory file with 200 ns to calculate the aggregated percentage of hydrogen bonds prevalence per HLA, using a Perl script plot_hbmap.pl provided by Dr. Justin Lemkul (https://www.thelemkullab.com/; last accessed 29 August 2023). In addition, the total number of hydrogen bonds as well other non-covalent contacts (Salt bridge, Pi-cation, Pi-stacking, T-stacking, Hydrophobic and van der Waals) between HLA-DRB1 alleles and the peptide were computed in the trajectory with GetContacts library (https://getcontacts.github.io/; last accessed 29 August 2023); and the interactions plotted as alluvial plots using an in-house-developed R script.

Binding energy scores were computed using AutoDock 4 [16,17] for a dataset of 1004 conformations. This ensemble of conformations was obtained by extracting 251 structures at 200 ps intervals from each of the four replicate trajectories of the studied pHLA systems. This analysis was conducted to assess the differences in binding affinity between citrullinated and native pHLAs. Results were presented as the mean binding affinity, accompanied by its standard deviation, both derived from the aforementioned ensemble of conformations.

### 4.4. Structures Visual Inspection

The 3D structures of pHLA complexes were visualized using PyMol v.2.4 software [31] and trajectories were visualized with VMD [37].

## Figures and Tables

**Table 1:** Vimentin peptide sequence information.

pHLA Name	Vimentin Length	Vimentin Sequence *
HLA-DRB1*04:01-vimetin71	66–78	SAVRL**R**SVPGVR
HLA-DRB1*04:01-vimetin71Cit	66–78	SAVRL**X**SSVPGVR
HLA-DRB1*04:04-vimetin71	66–78	SAVRL**R**SSVPGVR
HLA-DRB1*04:04-vimetin71Cit	66–78	SAVRL**X**SSVPGVR
HLA-DRB1*04:05-vimetin424	419–431	SSLNL**R**ETNLDSL
HLA-DRB1*04:05-vimetin424Cit	419–431	SSLNL**X**ETNLDSL

* The amino acid highlighted in bold represents the position in the sequence that interacts with P4 pocket in the shared epitope of the HLA. The “X” represents citrulline residue.

## Data Availability

The data used in this study are referenced throughout the text. Additional data will be made available by the authors upon request.

## Supplementary Materials

The following supporting information can be downloaded at: https://www.mdpi.com/article/10.3390/ijms26010034/s1.
