# Supplementary figures and images for: The Role of Vimentin Peptide Citrullination in the Structure and Dynamics of HLA-DRB1 Rheumatoid Arthritis Risk-Associated Alleles
Source: Int J Mol Sci. 2024 Dec 24;26(1):34. doi: 10.3390/ijms26010034 (PMC11719467; doi:10.3390/ijms26010034)

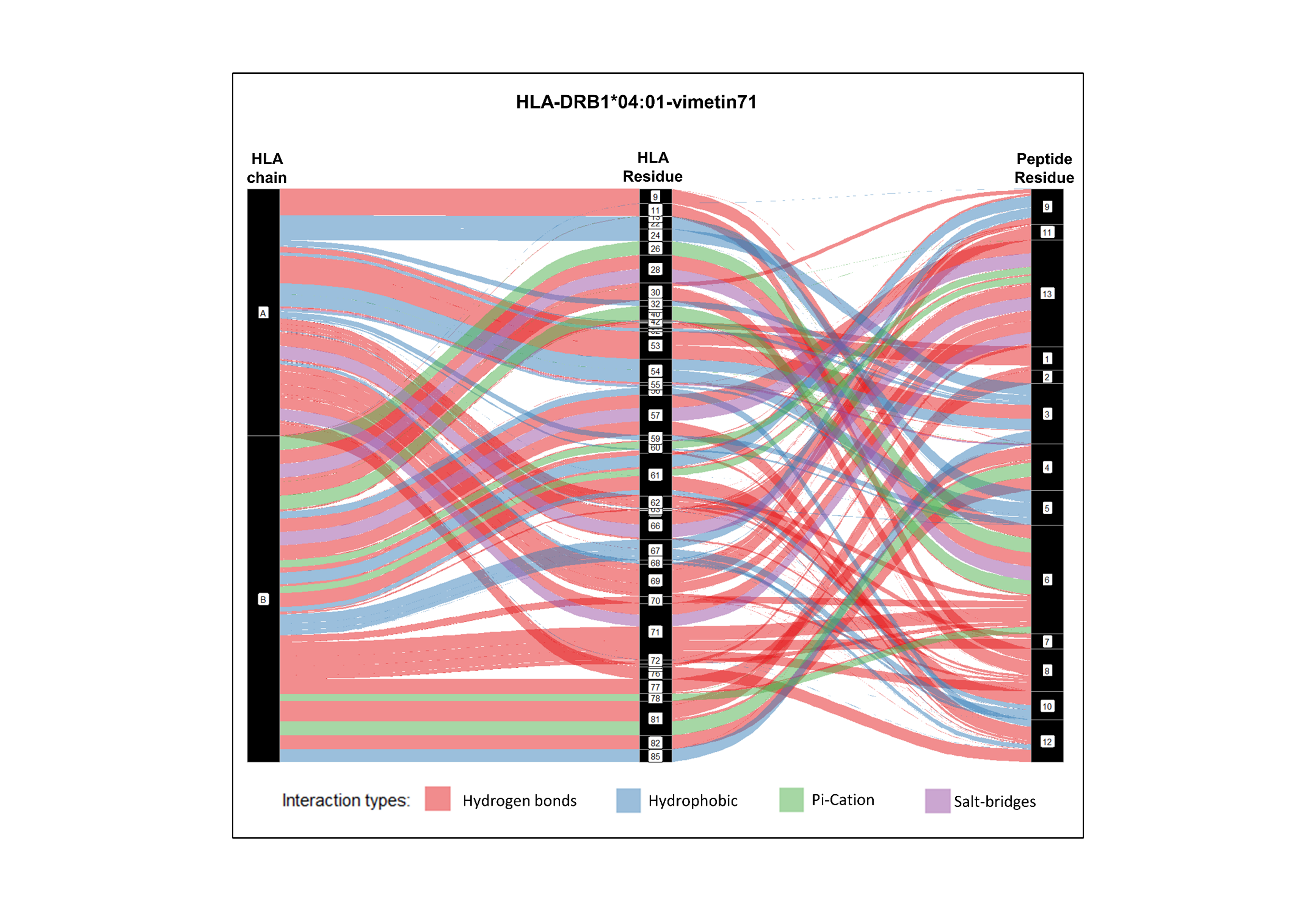

Supplement: Supplementary file 1 [file ijms-26-00034-s001.zip › FigureS1.png]

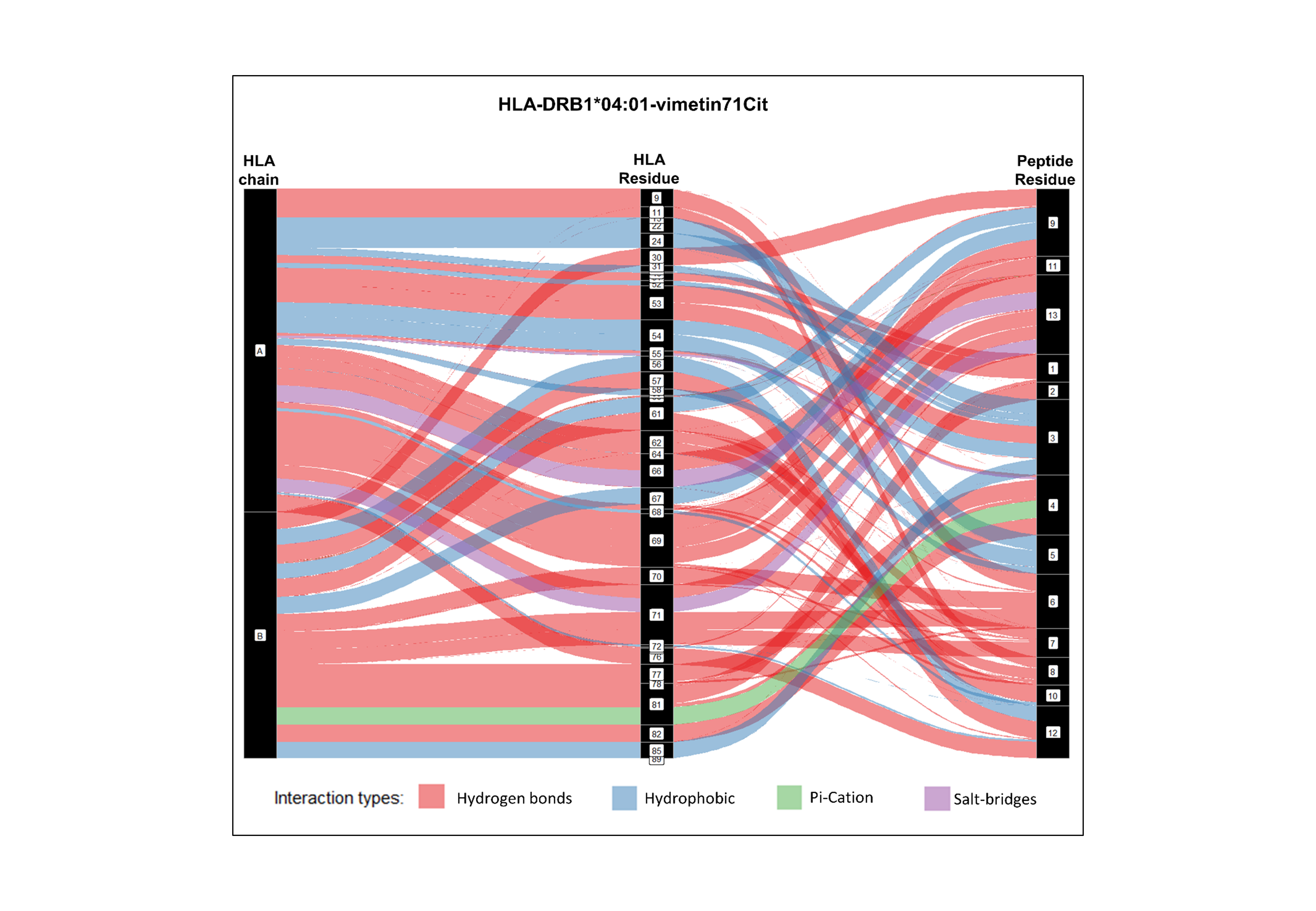

Supplement: Supplementary file 1 [file ijms-26-00034-s001.zip › FigureS2.png]

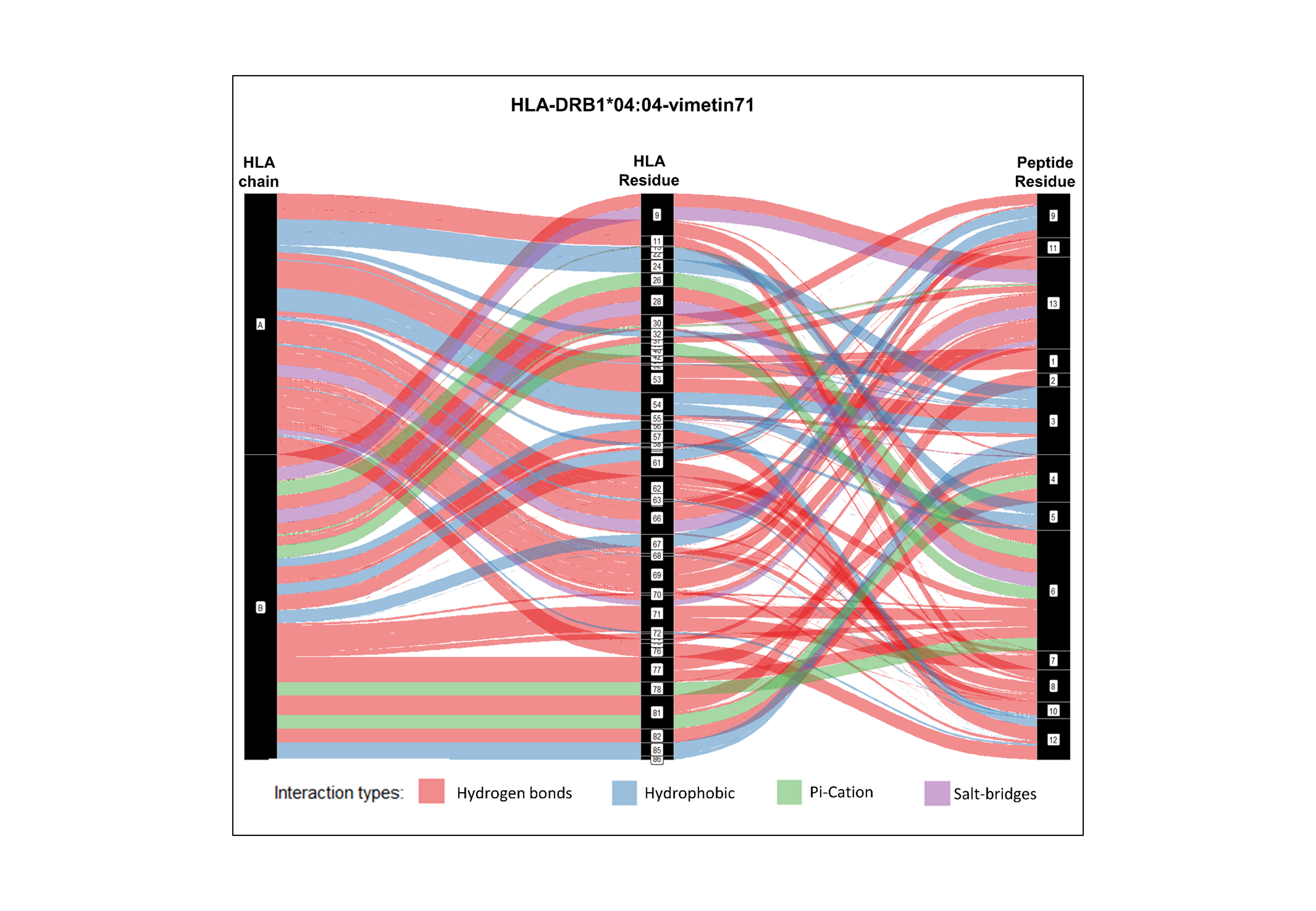

Supplement: Supplementary file 1 [file ijms-26-00034-s001.zip › FigureS3.png]

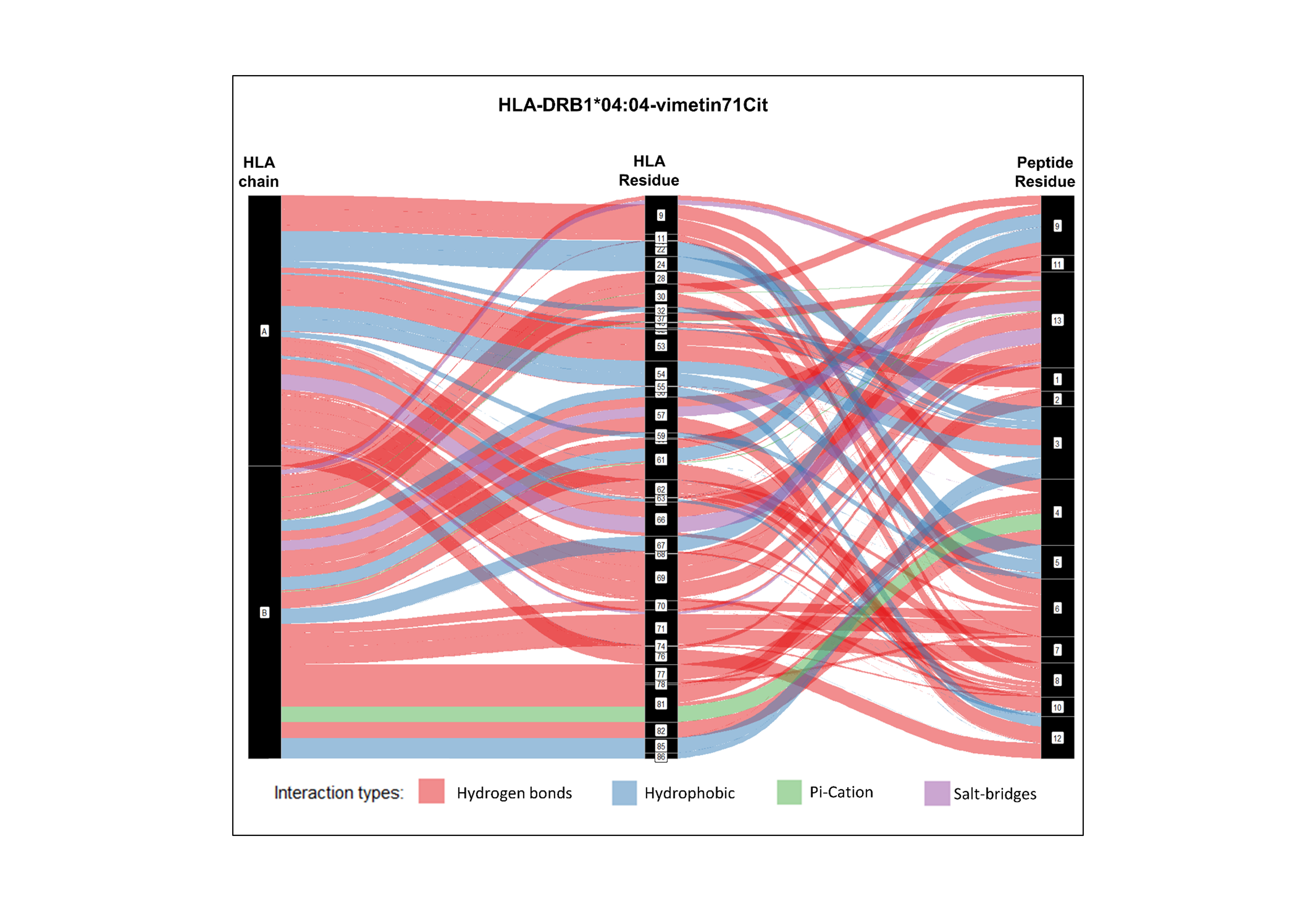

Supplement: Supplementary file 1 [file ijms-26-00034-s001.zip › FigureS4.png]

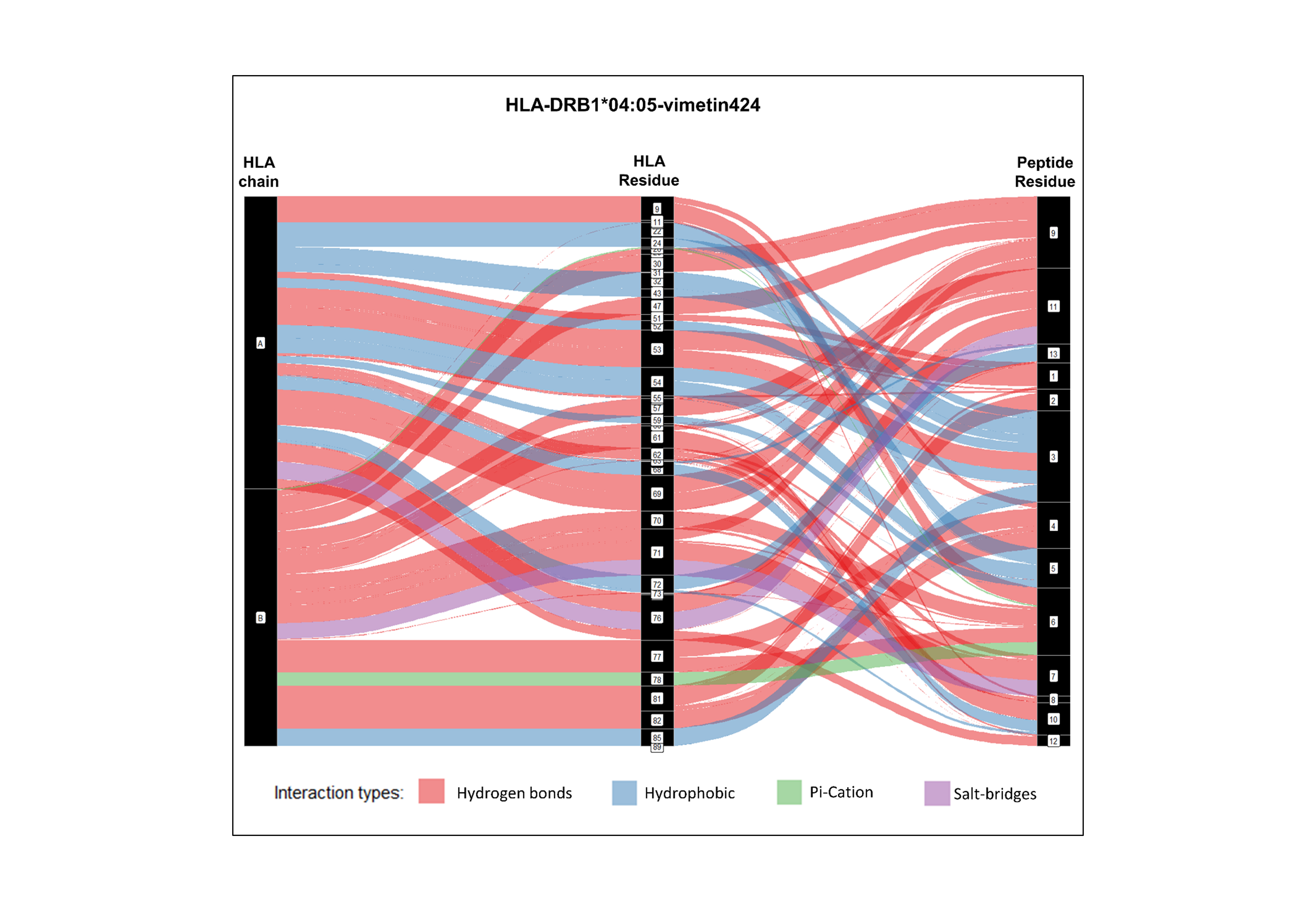

Supplement: Supplementary file 1 [file ijms-26-00034-s001.zip › FigureS5.png]

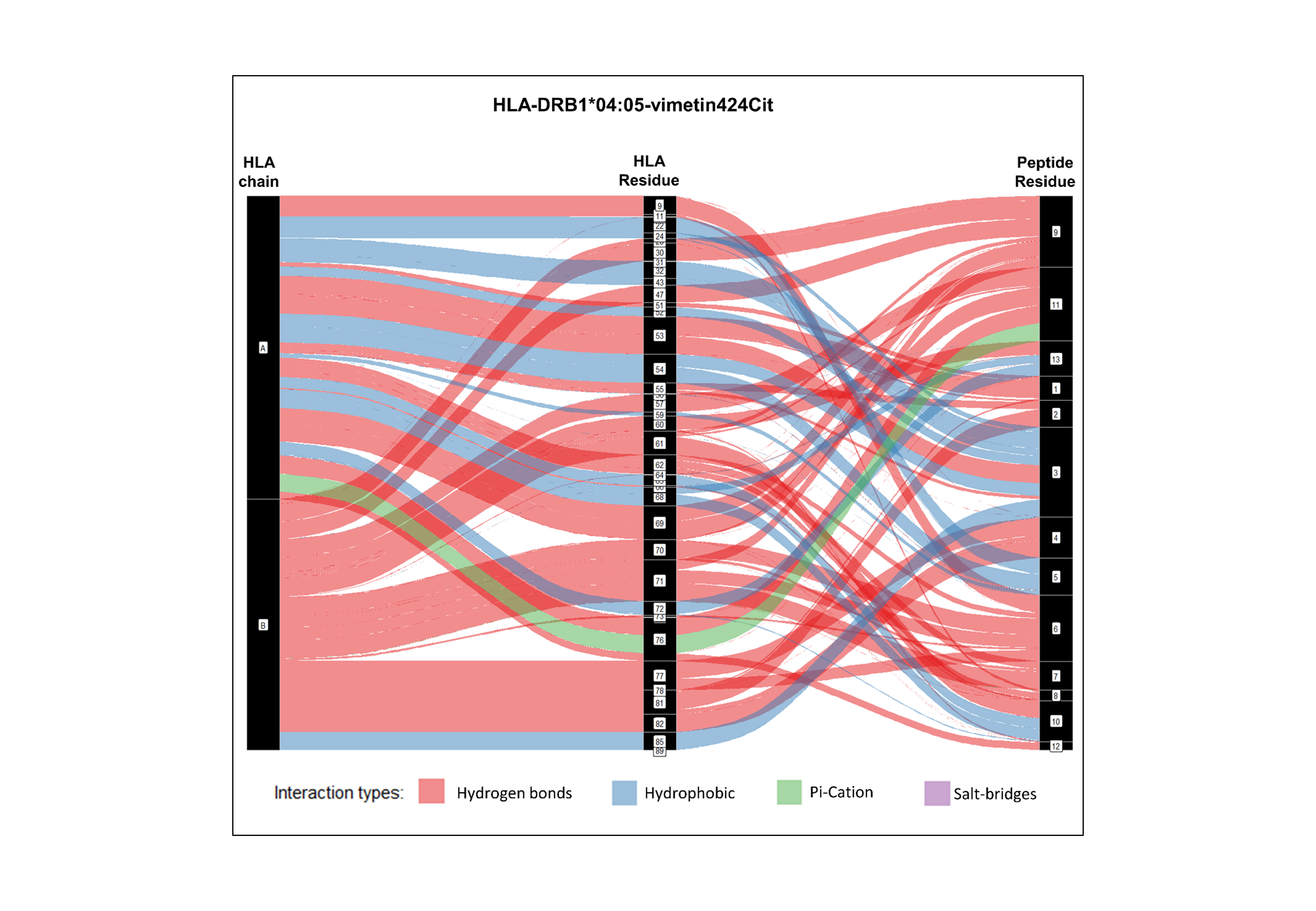

Supplement: Supplementary file 1 [file ijms-26-00034-s001.zip › FigureS6.png]
